# Prescription of Antibacterial Drugs for HIV-Exposed, Uninfected Infants, Malawi, 2004–2010 

**DOI:** 10.3201/eid2501.180782

**Published:** 2019-01

**Authors:** Alexander C. Ewing, Nicole L. Davis, Dumbani Kayira, Mina C. Hosseinipour, Charles van der Horst, Denise J. Jamieson, Athena P. Kourtis, for the Breastfeeding, Antiretrovirals and Nutrition study team

**Affiliations:** Centers for Disease Control and Prevention, Atlanta, Georgia, USA (A.C. Ewing, N.L. Davis, D.J. Jamieson, A.P. Kourtis);; University of North Carolina Project, Lilongwe, Malawi (D. Kayira);; University of North Carolina, Chapel Hill, North Carolina, USA (M.C. Hosseinipour, C. van der Horst)

**Keywords:** HIV-1, infant, drug resistance, antimicrobial resistance, antibacterial, Malawi, antibiotic stewardship, trimethoprim/sulfamethoxazole drug combination, infectious disease medicine, antibiotic prophylaxis, penicillins, poverty, HIV/AIDS

## Abstract

Antimicrobial drug resistance is a serious health hazard driven by overuse. Administration of antimicrobial drugs to HIV-exposed, uninfected infants, a population that is growing and at high risk for infection, is poorly studied. We therefore analyzed factors associated with antibacterial drug administration to HIV-exposed, uninfected infants during their first year of life. Our study population was 2,152 HIV-exposed, uninfected infants enrolled in the Breastfeeding, Antiretrovirals and Nutrition study in Lilongwe, Malawi, during 2004–2010. All infants were breastfed through 28 weeks of age. Antibacterial drugs were prescribed frequently (to 80% of infants), and most (67%) of the 5,329 prescriptions were for respiratory indications. Most commonly prescribed were penicillins (43%) and sulfonamides (23%). Factors associated with lower hazard for antibacterial drug prescription included receipt of cotrimoxazole preventive therapy, receipt of antiretroviral drugs, and increased age. Thus, cotrimoxazole preventive therapy may lead to fewer prescriptions for antibacterial drugs for these infants.

## Abstract 2

Cotrimoxazole preventive therapy may lead to fewer prescriptions.

The global rise of resistance to antibacterial drugs has resulted in longer illnesses, more deaths, and increased treatment costs (*1*–*4*). Although access to antibacterial drugs is more widespread in industrialized countries (*5*), low- and middle-income countries also experience the effects of antibacterial drug resistance and contribute to its spread (*6*). Because infants are more susceptible than adults to infectious diseases, antibacterial drug administration to infants is correspondingly higher (*7*,*8*). In sub-Saharan Africa, a growing population at especially high risk for infectious diseases is HIV-exposed, uninfected infants, many of whom breastfeed (*9*,*10*). 

We studied the healthcare needs and the magnitude of antibacterial use in such populations in an underresourced setting. We used longitudinal data about antibacterial drug prescribing for HIV-exposed, uninfected infants in a large randomized controlled trial of use of maternal and infant antiretroviral therapy (ART) to prevent mother-to-child transmission of HIV during breastfeeding, conducted in Malawi during 2004–2010. Our main objectives were to describe prescriptions for antibacterial drugs for HIV-uninfected infants, in terms of frequency of prescriptions, types of drug prescribed, and clinical indications for use; and to identify factors associated with increased hazard for prescription of antibacterial drugs for this population.

## Methods

### Study Enrollment, Design, and Procedures

Infants were enrolled in the Breastfeeding, Antiretrovirals and Nutrition (BAN) randomized clinical trial in Lilongwe, Malawi, during March 2004–January 2010 (*11*,*12*). The study enrolled 2,369 HIV-infected pregnant women >14 years of age with CD4+ counts of >250 cells/μL (>200 cells/μL before July 24, 2006) and their infants. Enrollment was limited to infants that weighed >2,000 g at birth and that had no condition precluding study interventions.

At the time of labor, all mothers received 1 dose (200 mg) of oral nevirapine followed by oral zidovudine (300 mg 2×/d) and lamivudine (150 mg 2×/d) for 7 days; their newborns received 1 dose of oral nevirapine (2 mg/kg bodyweight) and twice daily oral zidovudine (2 mg/kg bodyweight) and lamivudine (4 mg/kg) for 7 days. Using a factorial design, we randomly assigned eligible mother–infant pairs to receive or not receive a nutritional supplement while breastfeeding and to 1 of 3 ARV interventions to be initiated at birth and continued for 28 weeks or until breastfeeding cessation, if earlier. The ARV interventions were 1) daily nevirapine for the infant, 2) triple-drug ARV regimen for the mother, or 3) control (no treatment for mother or infant). According to a standardized protocol derived from the World Health Organization (WHO) Breastfeeding Counseling Training Manual (*13*), all mothers were individually counseled to breastfeed exclusively for the first 24 postpartum weeks and then wean rapidly during weeks 24–28. Because of overwhelming evidence of the intervention’s effectiveness, we stopped enrolling participants in the control group after we had 668 mother–infant pairs in this group; those already enrolled were offered the choice to switch to either of the interventions.

During the BAN study, WHO and the Malawi Ministry of Health released updated guidelines for prophylaxis for HIV-infected mothers and HIV-exposed infants. To adhere to these guidelines, daily cotrimoxazole preventive therapy (CPT) was implemented for mothers (480 mg 2×/d) and infants (240 mg 1×/d) enrolled in the study as of June 13, 2006, and for all those enrolled afterward. Infants began CPT at their first study visit after 6 weeks of age and continued through 36 weeks of age or until weaning was complete and HIV infection was ruled out.

At 2, 6, 12, 18, 24, 28, 36, and 48 weeks, we collected data on anthropometrics, vital signs, illnesses and hospitalizations since the last visit, current symptoms, and physical examination findings. Participants were advised to return to the clinic (to which they had unlimited access) between visits for treatment if the woman or child was ill. Medical care was provided according to the standard of care at the study clinics, and participants were given insecticide-treated bed nets. 

Guidelines for medication prescribing in Malawi are set by the Malawi Standard Treatment Guidelines, 4th Edition (*14*), and are based on the WHO Integrated Management of Childhood Illness Guidelines (http://whqlibdoc.who.int/publications/2005/9241546441.pdf). We collected data about prescriptions from the study concomitant medications log, which was abstracted from pharmacy records. If an infant had been examined by an outside healthcare provider, the mother was asked to report medications received. For hospitalized participating mothers and their infants, we obtained medical records when possible and included in our analysis any antibacterial drugs administered. At study visits, patients were asked if they had taken any medication not prescribed by study physicians, and any such medications were recorded. As outcomes, we considered only prescriptions for antibacterial agents (Table 1); other prescriptions, including antimalarial and antiparasitic medications, were excluded. Prescription of CPT was not considered an outcome.

**Table 1:** Antibacterial drugs used in the Breastfeeding, Antiretrovirals and Nutrition study, Malawi, 2004–2010

Class	Drug
Aminoglycoside	Gentamicin
Cephalosporins	Ceftriaxone
	Cefuroxime
	Loracarbef
Nitroimidazole	Metronidazole
	
Penicillins	Amoxicillin
	Ampicillin
	Augmentin
	Benzathine penicillin
	Benzylpenicillin
	Cloxacillin
	Flucloxacillin
β-lactams/β-lactamase inhibitor combination	Amoxicillin/clavulanate
Phenicols	Chloramphenicol
Quinolones	Ciprofloxacin
	Nalidixic acid
Sulfonamides	Cotrimoxazole
	Sulfadiazine
Tetracyclines/macrolides	Doxycycline
	Erythromycin
	Tetracycline

HIV status of infants at 2, 12, 28, and 48 weeks of age was determined by using a Roche Amplicor 1.5 DNA PCR (https://diagnostics.roche.com). Positive results were confirmed by testing an additional blood specimen. The window of infection was narrowed by testing dried blood spot specimens collected at 4, 6, 8, 18, 24, 32, and 36 weeks. We included in our analysis the 2,152 infants who were HIV negative at 2 weeks of age; we removed from the study and referred for care those who were not. 

### Ethics Approval

The BAN study was approved by the Malawi National Health Science Research Committee and the institutional review boards at the University of North Carolina at Chapel Hill and the US Centers for Disease Control and Prevention (CDC). All women provided written, informed consent for specimen storage and laboratory studies.

### Statistical Analyses

For descriptive analyses, we calculated frequencies and medians for all exposures and covariables. Total antibacterial drug prescriptions are presented as frequencies and as medians per infant and per infant-month of follow-up. We describe frequencies of prescriptions of antibacterials by drug class, indications, indication categories, respiratory indication subcategories, and routes of administration. We compare the categorical proportions of exposures, covariables, and total antibacterial drug prescriptions by using χ^2^ tests and assessed continuous variables by using the Kruskal–Wallis test. A Cox proportional hazards model with recurrent events modeled as a counting process was used to assess the hazards of antibacterial prescription by time-dependent CPT status; ARV group; malaria season (October–April); nutritional study group; maternal demographics; maternal CD4+ T-cell count at delivery; log maternal HIV viral load during pregnancy; and infant sex, birthweight, and categorical age (<1, 1–3, 3–6, or 6–12 mo). To assess the proportional hazards assumption and determine whether the effects of independent variables on the hazard of antibacterial prescription varied with infant age, we included interaction terms in Cox models. Infant follow-up ended at death, mother’s death, or loss to follow-up. For the 71 infants who became HIV infected after 2 weeks of age, follow-up ended at the time of their last HIV-negative test result. The first week of life and the 5 days after prescription of an antibacterial drug do not contribute to total follow-up time, and antibacterial drug prescriptions administered during these periods were excluded. Sensitivity analyses excluded the 71 infants who became HIV-infected after 2 weeks of follow-up and excluded prescriptions for topical antibacterials.

In analyses considering time-varying CPT exposure, infants were considered exposed from the first post-CPT implementation (June 13, 2006) study visit at or later than 6 weeks of age. Study group is modeled as an intent-to-treat variable. All analyses were performed by using SAS 9.4 (https://www.sas.com/).

## Results

At delivery, the mothers of the 2,152 infants included in this analysis had a median CD4+ T-cell count of 440 cells/μL and a median HIV viral load of ≈16,000 copies/mL (Table 2). Median age of the mothers was 26 years (interquartile range [IQR] 22–29 years), and most had been educated through the primary (53%) or secondary (34%) levels. Among infants, median birthweight was 3,000 g (IQR 2,700–3,300), and 51% were male. There were fewer infants in the control (28%) than the maternal ARV (35.6%) or infant nevirapine (36.7%) groups because of early discontinuation of the control group. Half of the mothers from all 3 groups received a nutritional supplement.

**Table 2:** Baseline characteristics of HIV-exposed, uninfected infants in the Breastfeeding, Antiretrovirals and Nutrition study, Malawi, 2004–2010*

Characteristic	Total	Before CPT	After CPT	p value†
No. infants	2,152	692 (32.16)	1,460 (67.84)	
Maternal education				1.0
None	245 (11.4)	81 (11.7)	164 (11.3)	
Primary	1,153 (53.7)	369 (53.3)	784 (53.8)	
Secondary	730 (34.0)	236 (34.1)	494 (33.9)	
Tertiary	21 (1.0)	6 (0.9)	15 (1.0)	
Maternal CD4, cells/μL‡	440 (330–582)	437 (328–596.5)	441 (330–578)	0.8
Maternal HIV viral load during pregnancy, copies/mL‡	16,045 (4,462–48,857)	17,231 (5,194.5–51,274)	15,281 (4,339–48,192)	0.2
Maternal age at delivery, y‡	26 (22, 29)	25 (22–29)	26 (23–29)	0.047
Male infant	1,088 (50.6)	358 (51.7)	730 (50.0)	0.5
Infant birthweight, g‡	3,000 (2,700–3,300)	3,000 (2,700–3,300)	3,000 (2,700–3,300)	0.7
Treatment group				0.002
Control	597 (27.7)	227 (32.8)	370 (25.3)	
Maternal ARV	766 (35.6)	229 (33.1)	537 (36.8)	
Infant nevirapine	789 (36.7)	236 (34.1)	553 (37.9)	
Nutrition group	1,076 (50.0)	345 (49.9)	731 (50.1)	0.9

*Data from 6 wks after birth overall and according to baseline visit timing relative to cotrimoxazole prophylaxis guideline implementation in June, 2006. Values are no. (%) unless otherwise indicated. ARV, antiretroviral therapy; CPT, cotrimoxazole preventive therapy.  †P values for continuous variables based on Kruskal-Wallis test.; p values for categorical variables based on the Pearson χ^2^ test. ‡Median (interquartile range).

**Figure 1:**
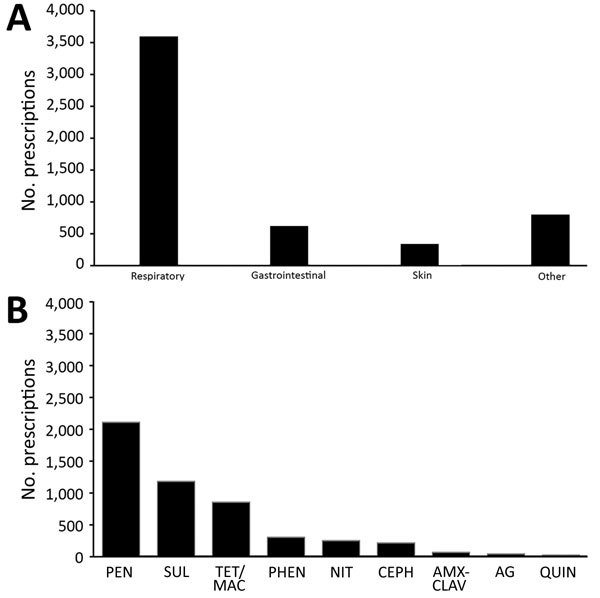
Numbers of prescriptions for antibacterial drugs, by clinical indication (A) and drug category (B), for HIV-exposed, uninfected infants in the Breastfeeding, Antiretrovirals and Nutrition study, Malawi, 2004–2010. AG, aminoglycosides; AMX-CLAV, amoxicillin-clavulanate; CEPH, cephalosporins; QUIN, quinolones; NIT, nitroimidazole; PEN, penicillins; PHEN, phenicols; SUL, sulfonamides; TET/MAC, tetracycline/macrolides.

Overall, 80.3% of infants received >1 prescription for an antibacterial drug during the follow-up period. Of the 5,107 antibacterial drug prescriptions for infants during the study, 3,437 (67.3%) were for respiratory indications, 590 (11.6%) for gastrointestinal indications, and 314 (6.1%) for skin-related indications (Figure 1, panel A). Among antibacterial drug prescriptions for respiratory indications, 66.7% were for acute respiratory infections or upper respiratory tract infections, 6.8% were for pneumonia, and 26.5% were for other conditions (Table 3). The distribution of respiratory indications among antibacterial drug prescriptions did not vary according to timing relative to CPT implementation. Most antibacterial drug prescriptions were for penicillins (2,132, 41.8%), sulfonamides (1,194, 23.4%), and tetracyclines or macrolides (863, 16.9%) (Figure 1, panel B). Each of the remaining categories (phenicols, nitroimidazoles, cephalosporins, β lactams/β-lactamase inhibitor combinations, aminoglycosides, and quinolones) accounted for <10% of prescriptions. In the BAN study, prescriptions for amoxicillin accounted for 1,932 (37.8%) of all antibacterial drug prescriptions for HIV-exposed, uninfected infants, followed by cotrimoxazole (23.4%), tetracycline (8.7%), erythromycin (8.2%), and chloramphenicol (6.0%). Tetracycline was prescribed as an ophthalmic solution to treat conjunctivitis. No other individual antibacterial drug accounted for >5% of all prescriptions (data not shown). Most antibacterial drugs were administered orally (85.0%); the rest, topically (8.0%) or intramuscularly (5.4%) (data not shown).

**Table 3:** Respiratory indications for antibacterial drugs prescribed for HIV-exposed, uninfected infants in the Breastfeeding, Antiretrovirals and Nutrition study, Malawi, 2004–2010*

Condition	No. (%)
Pneumonia	234 (6.81)
Acute respiratory infection or upper respiratory tract infection	2,291 (66.7)
Other*	912 (26.5)

*Otitis media, conjunctivitis, meningitis, sinusitis.

More infants were enrolled after (1,460) than before (692) CPT implementation in Malawi in June 2006 (Table 2). Median maternal age at delivery was slightly higher after CPT implementation (26 years) than before (25 years). Because of the discontinuation of the control group, infants born after CPT implementation were less likely to be in the control group (p<0.001 by χ^2^ test). No other maternal or infant characteristics differed according to CPT implementation or ARV treatment group (Table 2).

**Figure 2:**
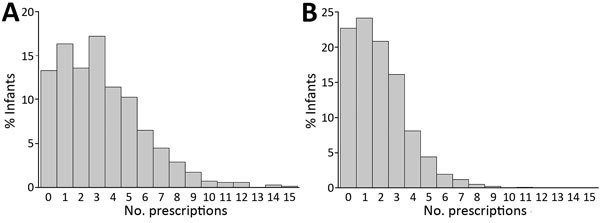
Distribution of HIV-exposed, uninfected infants according to total number of prescriptions for antibacterial drugs during follow-up in the Breastfeeding, Antiretrovirals and Nutrition study, Malawi, 2004–2010. Infants enrolled A) before and B) after implementation of cotrimoxazole preventive therapy.

Because more participants were enrolled after CPT implementation, the post-CPT implementation group contributed more person-time (412,697 vs. 187,865 person-days). The median length of follow-up for infants was 336 days (IQR 255–340 days); median follow-up length did not vary according to timing of birth relative to CPT implementation date (p = 0.09 by Kruskal–Wallis test) (Table 4). The median number of antibacterial drug prescriptions per infant was 2 (IQR 1–3) and was higher before (median 3, IQR 1–5) than after (median 2, IQR 1–3) CPT implementation. The same was true for median total antibacterial drug prescriptions per infant-month before (0.3, IQR 0.2–0.5) and after (0.2, IQR 0.1– 0.3) CPT implementation (Table 4). Several infants were born before CPT implementation, but only 1 was born after and received >10 antibacterial drug prescriptions over the follow-up period (Figure 2).

**Table 4:** Antibacterial prescriptions and person-time of follow-up for HIV-exposed, uninfected infants in the Breastfeeding, Antiretrovirals and Nutrition study, Malawi, 2004–2010*

Variable	Overall				Before CPT				After CPT				p values†	
Total no.	Per person	Per infant-month	Total no.	Per person	Per infant-month	Total no.	Per person	Per infant-month	Per person	Per person-month
No.	2,152				692				1,460					
Median follow-up time, d	600,562	336 (255–340)			187,865	337 (226–343)			412,697	336 (294–339)			0.09	
Prescriptions, total	5,107	2 (1–3)	0.2 (0.1–0.3)		2,269	3 (1–5)	0.3 (0.2–0.5)		28,38	2 (1–3)	0.2 (0.1–0.3)		<0.0001	<0.0001
For respiratory infections	3,437	1 (0–2)	0.1 (0–0.2)		1481	2 (1–3)	0.2 (0.1–0.3)		1956	1 (0–2)	0.1 (0.0–0.2)		<0.0001	<0.0001
For other infections	1,670	0 (0–1)	0 (0–0.1)		788	1 (0–2)	0.1 (0–0.2)		882	0 (0–1)	0.0 (0.0–0.1)		<0.0001	<0.0001

*Overall and according to baseline visit timing relative to CPT guideline implementation (June, 2006). Values are range (IQR) except as indicated. CPT, cotrimoxazole prophylaxis; IQR, interquartile range. †p values based on Kruskal-Wallis test for continuous variables.

Several factors were significantly associated with antibacterial drug prescription according to the Cox proportional hazards model. The largest reduction in hazards of prescription was for time-varying CPT exposure (hazard ratio [HR] 0.57, 95% CI 0.52–0.61) (Table 5). Assignment to each of the ARV prophylaxis groups was also associated with reduced hazard of antibacterial drug prescription (maternal ARV HR 0.85, 95% CI 0.78–0.93; infant nevirapine HR 0.90, 95% CI 0.82–0.98). Although maternal CD4+ counts at delivery were not associated with antibacterial drug prescriptions, log maternal HIV viral load at delivery was; for each 1 unit increase of log maternal viral load, hazard of infant antibacterial drug prescriptions increased by 2% (HR 1.02, 95% CI 1.003–1.04).

**Table 5:** Cox proportional hazard ratio estimates for associations between prescriptions for antibacterial drugs and other variables in the Breastfeeding, Antiretrovirals and Nutrition study, Malawi 2004–2010

Factor	Hazard ratio (95% CI)
Cotrimoxazole preventive therapy	0.57 (0.52–0.61)
Malaria season (Oct–Apr)	0.98 (0.91–1.05)
Treatment group	
Maternal antiretrovirals	0.85 (0.78–0.93)
Infant nevirapine	0.90 (0.82–0.98)
Nutritional supplement	1.05 (0.98–1.12)
Maternal CD4+ T-cell count at delivery, cells/μL	1.15 (0.96–1.38)
Maternal HIV viral load during pregnancy, log copies/mL	1.02 (1.003–1.04)
Maternal age, y	1.01 (0.998–1.01)
Maternal education	
None	Reference
Primary	0.92 (0.83–1.03)
Secondary	0.99 (0.89–1.11)
Tertiary	0.93 (0.65–1.32)
Male sex	1.09 (1.02–1.17)
Infant birthweight, kg	1.17 (1.06–1.28)
Age category, mo	
Birth–1 mo	Reference
1–3	0.80 (0.67–0.95)
3–6	0.63 (0.53–0.76)
6–12	0.48 (0.40–0.58)

Removing the 71 infants who had become HIV infected by the 2-week visit reduced the median maternal viral load for CPT-exposed and CPT-unexposed infants but did not otherwise notably affect results. Exclusion of topical antibacterial drug prescriptions did not alter any associations in the proportional hazards model (results not shown).

No maternal demographic characteristics were associated with hazard of antibacterial drug prescription, but infant characteristics were associated. Male sex (HR 1.09, 95% CI 1.02–1.17) and higher birthweight (HR 1.17, 95% CI 1.06–1.28) were each associated with increased hazard of antibacterial drug prescriptions; increasing age was associated with reduced hazard of prescriptions. Compared with the period from birth to 1 month of age, each subsequent period was associated with further reduced hazard of antibacterial drug prescription (at 1–3 months of age, HR 0.80, 95% CI 0.67, 0.95; at 3–6 months, HR 0.63, 95% CI 0.53, 0.76; at 6–12 months, HR 0.48, 95% CI 0.40, 0.58) (Table 5).

## Discussion

In this study, HIV-exposed uninfected infants in Lilongwe, Malawi, received a median of 2 antibacterial drug prescriptions during the 48 weeks of study follow-up; 80% received >1 prescription during their first year of life. The main indicator driving these prescriptions was respiratory infections, followed by gastrointestinal infections. The most commonly prescribed drugs were in the penicillin class, specifically amoxicillin. Prescriptions became less common as study infants aged, and prophylactic CPT was associated with a 43% decrease in antibacterial drug prescriptions to treat infections; ARV administration to either the mother or the infant was associated with smaller decreases (10%–15%).

Studies of recent antibacterial drug prescribing in western Europe and Australia show rates of 0.65–0.97 prescriptions/person-year for infants in the first year of life (*15*–*19*), approximately one third to one half the rate of prescriptions for infants in the BAN study. This finding is perhaps not surprising, considering the higher rates of illness and death caused by infectious diseases among infants in Malawi compared with rates in higher income countries (*20*) and the even higher illness rates for infants born to HIV-infected mothers (*9*). Previous research has identified irrational prescribing of antibacterial drugs in health facilities in Malawi (*21*), potentially contributing to the higher observed rates of antibacterial drug use. To our knowledge, studies using person-level data to determine antibacterial drug prescription for infants in low- and middle-income countries are lacking. Antibacterial drugs were prescribed for a larger proportion (80%) of infants in the BAN study than in a study in Nigeria (*22*), although that study was based on parent recall, which may have introduced error.

CPT for participants in the BAN study was implemented in June 2006. Several analyses, using data from the BAN study and from other studies, have shown CPT to be associated with reduced subclinical malaria (*23*), lower rates of illness and death caused by infectious diseases (*24*–*26*), and reduced incidence of severe anemia (*27*). The antibacterial properties of cotrimoxazole are probably the cause of the observed association between CPT and reduced overall antibacterial prescriptions. The malaria-preventing activity of cotrimoxazole may also contribute to better overall infant health and decreased illness rates from infectious diseases, as indicated in another analysis of BAN data (*24*). The prescriptions in this trial were driven mostly by respiratory infections. Reductions in antibacterial drug prescriptions associated with gastrointestinal symptoms and other indications were also observed; this finding is in agreement with previous results from the BAN study showing associations between CPT and reduced respiratory and diarrheal illness (*24*) and with evidence that CPT prevents a variety of infections and resulting illness and death (*28*–*30*). Another study, however, found that CPT for severely malnourished infants in Kenya did not reduce respiratory or diarrheal infections, although it did reduce malaria incidence (*31*).

Significant reductions in antibacterial drug prescriptions, although of smaller magnitude, were associated with use of ART by mothers during breastfeeding and with prophylactic administration of nevirapine to infants during 28 weeks of breastfeeding. Factors contributing to such increases may include overall improvements in maternal health, resulting in better ability to care for their infants, and perhaps improved immunologic or nutritional quality of breastmilk (*32*,*33*). The reasons for the effect of nevirapine on the infant are less clear but may be associated with the mother’s perception of her infant’s health and need to access care. Since the conclusion of the BAN study, Malawi has adopted the Option B+ strategy (http://www.who.int/hiv/pub/guidelines/arv2013/en) aiming to provide all HIV-infected persons with ART for life. Guidelines for HIV-infected pregnant women (https://aidsfree.usaid.gov/sites/default/files/malawi_art_2016.pdf) have been updated to recommend that mothers continue breastfeeding for 12–24 months and continue CPT, both of which may lead to decreased infectious disease incidence for the infants and resultant decreased prescription of antibacterial drugs.

Among antibacterial drugs given for respiratory indications, approximately two thirds were for acute respiratory infections or upper respiratory tract infections, for which Malawi Ministry of Health guidelines (http://www.pascar.org/uploads/files/Malawi_STG_Hypertension.pdf) do not support antibacterial treatment. This pattern agrees with research on prescribing practices from Malawi and other developing countries (*21*,*34*,*35*) but also the United States (*36*,*37*). After implementation of CPT, the prescriptions described above and appropriate prescription of antibacterial drugs declined, which could possibly be attributed to reductions of malaria or better overall maternal and infant health. In addition, recent research indicates that pharmacies in Malawi often do not adhere to rules requiring a prescription for antibacterial drugs (*38*), although there are no data on administration of over-the-counter antibacterial drugs for infants. The BAN study attempted to record these medications, but the self-reported nature of these data could have led to underestimation.

Maternal HIV viral load is an indicator of HIV disease status and an indirect marker of immune status; higher viral loads indicate more advanced HIV disease, a more compromised immune system, and increased susceptibility to infection (*39*). Mothers with a higher viral load at delivery may have had poorer overall health and increased susceptibility to infection, potentially explaining the increase in antibacterial drug prescriptions for infants of mothers with higher viral loads. The BAN study excluded women with CD4+ counts <250 cells/μL (<200 cells/μL early in the study). This restriction may explain why no association was found between CD4^+^ counts and antibacterial drug prescriptions.

Our findings also showed an association of increased antibacterial drug prescriptions for infants that were male, had higher birthweight, and were younger. Research suggests that male infants are more susceptible to most types of respiratory infections and that these infections tend to be more severe (*40*). Younger infants also have greater susceptibility to, and severity of, infectious diseases (*8*,*41*,*42*), which could trigger more aggressive treatment of younger infants. The reasons for the association of antibacterial drug prescriptions with higher birthweight are less clear; however, infants with the lowest birthweights (<2,000 g) were excluded from the BAN study.

The 13-valent pneumococcal conjugate vaccine was introduced in Malawi in November 2011, after the conclusion of the BAN study. Surveillance during implementation of this immunization program showed fewer cases of severe and fatal pneumonia (*43*). This vaccine can also prevent otitis media caused by pneumococcal infection (*44*). The rotavirus vaccine, introduced in October 2012, also after the conclusion of the BAN study, has reduced hospital admissions of infants for acute rotavirus gastroenteritis in Malawi (*45*). The reduction of severe pneumonia may reduce infants’ need for antibacterial drugs, and the reduction of rotavirus infections may limit unnecessary antibacterial drug prescribing and generally improve infant health.

Our study has some limitations. One limitation is a lack of reliable data about the duration of treatment with antibacterial drugs and individual adherence. Although the study followed Malawi national treatment guidelines, as part of a large clinical trial that provided unlimited access to medical care, resources may have been available to the study clinics that are not available to clinics and persons at large in the target population in Malawi, limiting our ability to generalize the results. Also, we were not able to independently verify the reliability of providers’ diagnoses used to guide prescription of antibacterial drugs. Although we requested relevant records when participants reported having received outside medical care, some antibacterial drug prescriptions may have been missed. However, because medical care was provided without charge, participants had an incentive to use that provided by the study clinic. 

There may have been unmeasured variables affecting disease incidence or antibacterial drug prescribing patterns, confounding the associations we found. Because of its time-dependent nature, the association between CPT exposure and antibacterial drug use may be vulnerable to confounding by overall trends in population health. However, baseline characteristics before and after CPT implementation were broadly comparable. Prescriptions for antibacterial drugs when such treatment was not clearly indicated (e.g., fever, abdominal pain, upper respiratory tract infections, asthma) were included as outcomes. For these conditions, this study lacks sufficient clinical detail on which to base a definite determination of the appropriateness of antibacterial drug administration, and we thus cannot definitely conclude how much of the reduced antibacterial drug prescription associated with CPT resulted from prevention of bacterial infections versus other factors, such as reduced malaria or influence on clinicians’ antibacterial drug prescribing threshold. A proportion of the reduced antibacterial drug prescriptions for respiratory indications associated with CPT may result from a reduction in malaria because of overlapping symptoms (*46*,*47*). In Malawi, because study clinicians commonly do not regularly test urine without a distinct indication of a urinary tract problem, urinary tract infections are probably underrepresented as causes for prescription of antibacterial drugs in the study population.

Our study also has several strengths. The study included a large sample of a population whose antibacterial drug use is underdescribed, it took advantage of the mid-trial CPT introduction to assess CPT effects on use of antibacterial drugs, and it measured the effects of several factors on prescribing of antibacterial drugs for HIV-exposed, uninfected infants in an urban setting in Malawi. Data on independent variables, outcomes, and illness were collected systematically, and participants were followed up actively, which is generally not possible with studies that use administrative or surveillance data.

In conclusion, our findings indicate that antibacterial drugs were frequently prescribed for HIV-exposed, uninfected infants in the BAN study, primarily to treat respiratory infections. Antibacterial drugs were more likely to be prescribed for infants who were younger, whose mothers had higher HIV viral loads, and who were not exposed to ART. We found a strong association between CPT and lower hazard of antibacterial drug prescription. With the expansion of lifelong ART coverage in Malawi and other areas of high HIV prevalence and the increasing availability of effective vaccines, HIV-exposed, uninfected infants in Malawi may experience fewer infectious diseases and a resulting decrease in prescription of antibacterial drugs. Our study provides a useful reference point for measuring prescription of antibacterial drugs for infants at high risk for infectious diseases in a low-income country and for assessing the effects of programs to improve the health of those infants in Malawi.
